# PheW^2^P2V: a phenome-wide prediction framework with weighted patient representations using electronic health records

**DOI:** 10.1093/jamiaopen/ooae084

**Published:** 2024-09-14

**Authors:** Jia Guo, Krzysztof Kiryluk, Shuang Wang

**Affiliations:** Department of Biostatistics, Columbia University, New York, NY 10032, United States; Department of Medicine, Columbia University, New York, NY 10032, United States; Department of Biostatistics, Columbia University, New York, NY 10032, United States

**Keywords:** phenome-wide prediction, patient representations, electronic health records (EHRs)

## Abstract

**Objective:**

Electronic health records (EHRs) provide opportunities for the development of computable predictive tools. Conventional machine learning methods and deep learning methods have been widely used for this task, with the approach of usually designing one tool for one clinical outcome. Here we developed PheW^2^P2V, a *Phe*nome-*W*ide prediction framework using *W*eighted *P*atient *V*ectors. PheW^2^P2V conducts tailored predictions for phenome-wide phenotypes using numeric representations of patients’ past medical records weighted based on their similarities with individual phenotypes.

**Materials and Methods:**

PheW^2^P2V defines clinical disease phenotypes using Phecode mapping based on International Classification of Disease codes, which reduces redundancy and case-control misclassification in real-life EHR datasets. Through upweighting medical records of patients that are more relevant to a phenotype of interest in calculating patient vectors, PheW^2^P2V achieves tailored incidence risk prediction of a phenotype. The calculation of weighted patient vectors is computationally efficient, and the weighting mechanism ensures tailored predictions across the phenome. We evaluated prediction performance of PheW^2^P2V and baseline methods with simulation studies and clinical applications using the MIMIC-III database.

**Results:**

Across 942 phenome-wide predictions using the MIMIC-III database, PheW^2^P2V has median area under the receiver operating characteristic curve (AUC-ROC) 0.74 (baseline methods have values ≤0.72), median max F_1_-score 0.20 (baseline methods have values ≤0.19), and median area under the precision-recall curve (AUC-PR) 0.10 (baseline methods have values ≤0.10).

**Discussion:**

PheW^2^P2V can predict phenotypes efficiently by using medical concept embeddings and upweighting relevant past medical histories. By leveraging both labeled and unlabeled data, PheW^2^P2V reduces overfitting and improves predictions for rare phenotypes, making it a useful screening tool for early diagnosis of high-risk conditions, though further research is needed to assess the transferability of embeddings across different databases.

**Conclusions:**

PheW^2^P2V is fast, flexible, and has superior prediction performance for many clinical disease phenotypes across the phenome of the MIMIC-III database compared to that of several popular baseline methods.

## Introduction

The increasing adoption of electronic health records (EHRs) brings opportunities to develop new computational predictive tools.^1–3^ Conventional machine learning approaches such as regression-based, bagging, or boosting methods have been widely used to predict clinical outcomes such as heart failure, type 2 diabetes mellitus, hypertension, and others.^4–8^ We recently developed a flexible similarity-based algorithm and applied it to predict end stage kidney disease and severe aortic stenosis.^9^ With these conventional methods, usually one prediction tool is trained for one outcome, that is, they are outcome-specific, and only labeled data are used to train the model, that is, they are fully supervised. In addition, these conventional methods usually take data that are well-structured without missing values.

Deep learning algorithms for natural language processing (NLP) have also been used for clinical decision making with EHR, because patients’ sequences of medical records are similar to sequences of words in text documents. Historically developed for NLP tasks such as machine translation to fully use sequence information, recurrent neural network (RNN)^10^^,^^11^ has been widely used in EHR to predict health outcomes. With the word2vec algorithm being introduced^12^ in 2013, which represents words with numeric vectors, medical records embeddings can be pre-trained and combined with prediction models such as RNN or logistic models, either in a 2-step fashion without fine-tuning embeddings, that is, frozen embeddings plus prediction, or in a 1-step fashion that fine-tunes embeddings based on outcomes. In a 1-step approach, RNN models fine tune pre-trained embeddings together with RNN parameters to predict outcomes such as clinical diagnoses or readmissions.^13–16^ Thus, 1-step models are outcome-specific, fully-supervised, and computationally intensive. On the other hand, 2-step models are not outcome-specific, not fully supervised as unlabeled data can be used for embedding and embedding is done once and is combined with a prediction model to predict outcomes. Two-step models are thus computationally efficient. Farhan et al proposed a 2-step model,^17^ where a patient’s sequence of medical records was numerically represented by summing up their medical concept embeddings and was subsequently used to predict the patient’s risks of different diagnoses. However, the prediction performance is only slightly better than that of logistic regressions because all medical records were treated equally regardless of the diagnoses to be predicted.

To improve prediction performance and computational efficiency, the Transformer model^18^ was developed, which uses a position embedding and self-attention layers to capture relative contributions of words in a sentence and conducts predictions in a 1-step fashion. BERT (Bidirectional Encoder Representations from Transformers)^19^ was subsequently developed to improve Transformer through pre-training a Transformer encoder by predicting randomly masked words. BERT can be combined with different prediction models, such as RNNs or Transformer decoders, either in a 2-step fashion, or a 1-step fashion. BERT has also been applied in EHR. The Med-BERT model^20^ was pre-trained on a large external dataset with 28 million patients, and the model was fine-tuned using RNN to predict 2 diseases, heart failure among diabetic patients and onset of pancreatic cancer. However, the prediction gains of Med-BERT through pre-training using such a large external dataset are minimal compared to the computational cost.

Here we aim to conduct phenome-wide predictions with computational efficiency while maintain good prediction performance for individual phenotypes. The prospective phenome-wide predictions could be useful as a screening tool to flag patients with high-risk conditions in early stages which may be missed overwise. Specifically, we propose PheW^2^P2V, a *Phe*nome-*W*ide prediction framework that uses *W*eighted *P*atient *V*ectors. PheW^2^P2V is the first phenome-wide prediction framework that takes a 2-step procedure, that is, embedding plus prediction, thus is computational efficient. To maintain good prediction performance for individual phenotypes, PheW^2^P2V uses a novel weighting scheme on medical concept embeddings so that predictions based on patient embeddings are tailored to individual phenotypes. Since diagnosis concepts in EHR are usually coded using International Classification of Disease (ICD) terminology, which is designed for billing and administrative functions but not for case-control studies,^21^ PheW^2^P2V first maps patients’ ICD codes to clinical disease phenotypes called phenotype codes (phecodes). Phecodes are originally developed for phenome-wide association studies (PheWAS), where patients’ phenotypes are identified by grouping ICD codes that represent common etiologies, with a purpose of reducing the redundancy and better defining cases and controls.^21^^,^^22^ To predict a clinical disease phenotype in the phenome, after generating medical concepts embeddings using word2vec, PheW^2^P2V represents each patient as a single numeric patient vector where patient’s medical concepts that are more correlated with the phenotype of interest are upweighted. The patient vector is then used to predict the incidence risk of the phenotype of the patient.

Unlike the 1-step model where embeddings are fine-tuned for one outcome of interest, PheW^2^P2V introduces weights on medical concepts to improve computational efficiency while tailors predictions to a phenotype of interest to maintain good phenome-wide prediction performance. Unlike traditional supervised methods that only use labeled data to supervise predictions, PheW^2^P2V uses labeled and unlabeled training samples for embeddings, which are then used for predictions. This can mitigate the overfitting problem that most supervised methods encounter.

Using simulation studies, we showed an improved prediction of PheW^2^P2V over that of 4 baseline methods including a regression-based model, a random forest classifier, a gradient boosted tree classifier, and the P2V model without weights.^17^ We applied all methods to the MIMIC-III database^23^ to predict patients’ incidence risks of 942 phenotypes at the latest visit using medical records from past visits. We observed better predictions of PheW^2^P2V consistently across most phenotypes over that of baseline methods. We also demonstrated several clinical examples in which PheW^2^P2V can predict less-common conditions that could be diagnostically challenging or missed on a routine clinical work up, such as chronic pericarditis. Automated suggestions provided by PheW^2^P2V that such conditions should be considered in the differential diagnosis and in the work up of high-risk patients could be clinically impactful.

## Methods

### Overview of PheW^2^P2V

**Figure 1.**
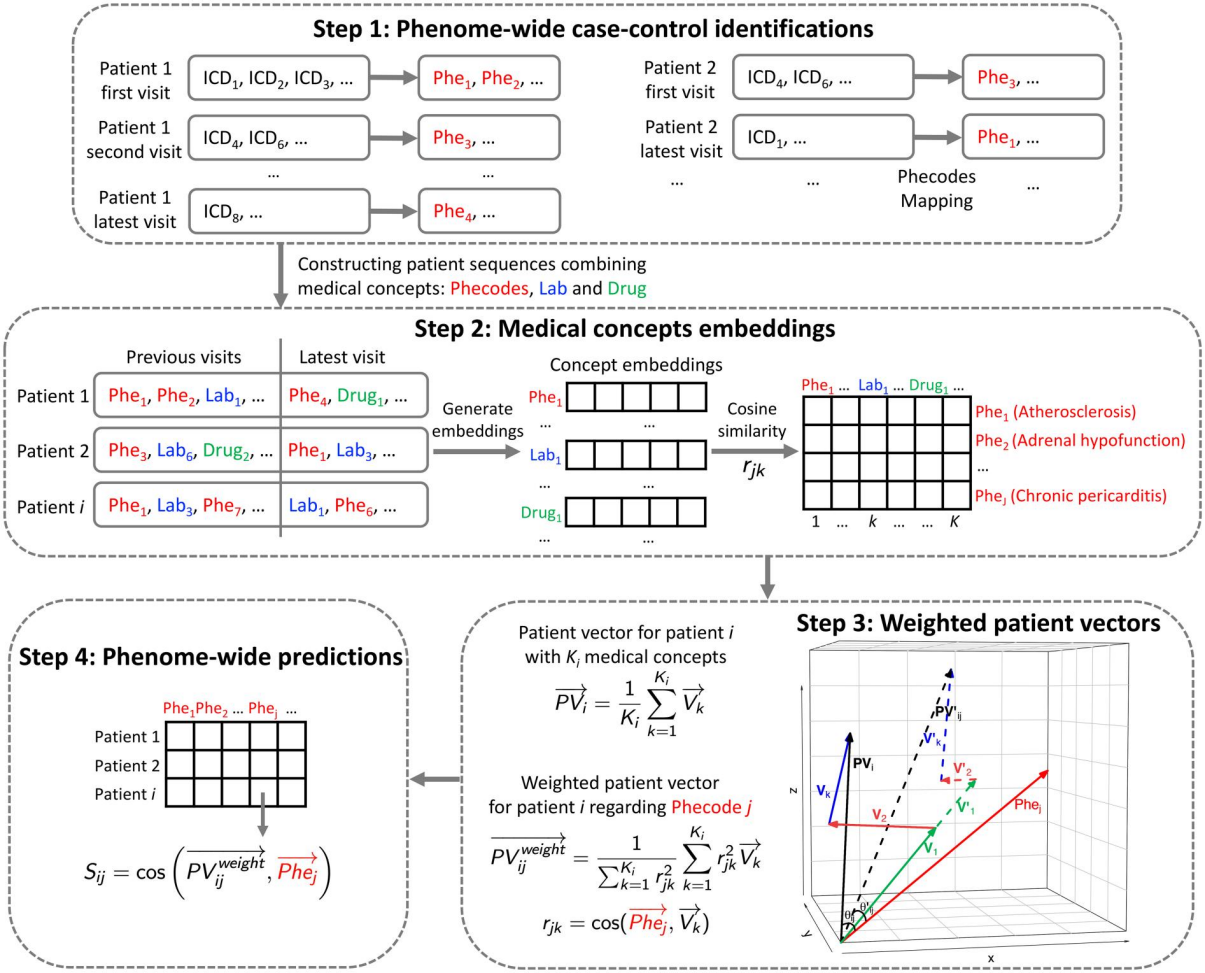
The workflow of the proposed PheW^2^P2V framework.

The PheW^2^P2V framework is illustrated in Figure 1 with 4 steps: (1) identifying case-control status of phenome-wide clinical disease phenotypes by mapping diagnosis ICD codes to phenotype codes (phecodes) and constructing patient sequences; (2) generating medical concepts embeddings using word2vec; (3) calculating weighted patient vectors with weights capturing correlations between past medical records and a phenotype of interest; and (4) conducting tailored phenome-wide predictions using weighted patient vectors to predict incidence risks of individual patients.

### Step 1: phenome-wide case-control identifications

Phenotype mapping using Phecode map^21^ (with R package “PheWAS”^24^) aims to reduce the redundancy of ICD codes and more accurately define case-control status of clinical phenotypes, for the purpose of phenome-wide analysis. With phecode mapping, over 14 000 codes in the ICD-9 system were grouped into approximately 1600 phenotype codes (phecodes).^22^ Using the MIMIC-III database, for each patient’s visit, PheW^2^P2V maps patient’s ICD-9 codes to phecode-defined “case groups,” that is, case status of meaningful clinical phenotypes. A list of disease-specific exclusion phecodes was also specified for each “case group.” A patient without any ICD-9 code in this list is defined as the “control group.” This mapping ensures that patients with comparable diseases are not categorized as controls. For example, a patient with an unknown arrhythmia cannot be considered as a control for atrial fibrillation.^24^

### Step 2: medical concepts embeddings

Word2vec is a NL embedding algorithm that is computationally efficient.^12^ With a large corpus of text, it uses a neural network to generate numeric vectors for unique words in the corpus. These numeric vectors have the same dimension and are thus embedded in a vector space. A patient’s sequence of medical concepts that contains phenotype codes, lab test codes, and medication codes can be considered as a sentence with words. PheW^2^P2V applies word2vec to learn numeric vectors for medical concepts. The clinical similarity between 2 medical concepts can be captured by the cosine similarity between the 2 corresponding numeric vectors.

### Step 3: weighted patient vectors tailored for a phenotype

For each phenotype, we calculate cosine similarities between the numeric vector of the phenotype and numeric vectors of all other medical concepts. Suppose an EHR database has *K* unique medical concepts, among which there are *J* phenotype codes. We build a correlation matrix with dimension *J × K* to capture correlations between *J* phenotypes and *K* medical concepts. To conduct tailored predictions, a patient’s past medical records are summarized into a numeric patient vector, which is a weighted average of numeric vectors of the patient’s past medical concepts using phenotype-specific weights to up-weight medical concepts that are most relevant to the phenotype:

#(1) PVijweight→=1∑k=1Kirjk2∑k=1Kirjk2Vk→,rjk=cosinePhej→,Vk→.

Here we calculate the weighted patient vector $\vec{{\mathrm{PV}}_{ij}^{\mathrm{weight}}}$ for patient *i* tailored for phenotype *j*, where $K_{i}$ is the number of concepts of patient *i*, $\vec{V_{k}}$ is the vector representation of medical concept *k*, $\vec{{\mathrm{Phe}}_{j}}$ is the vector representation of phenotype *j*, and $r_{jk}$ is the cosine similarity between medical concept *k* $\vec{V_{k}}$ and phenotype *j* $\vec{{\mathrm{Phe}}_{j}}$. The weight of medical concept *k* tailored for phenotype *j* for patient *i* is defined as $\frac{r_{jk}^{2}}{\sum_{k=1}^{K_{i}}r_{jk}^{2}}$, where we treat negative and positive correlations equally. We note that a negative $r_{jk}$ suggests a negative association between medical concept *k* and phenotype *j*, which helps prediction similarly as a positive association.

### Step 4: phenome-wide risk predictions

To predict risk of phenotype *j* for a test sample *t*, we compute the test sample’s patient vector $\vec{{\mathrm{PV}}_{tj}^{\mathrm{weight}}}$ using eqn (1) and the following risk score, which is the cosine similarity between the patient vector and the phenotype vector $\vec{{\mathrm{Phe}}_{j}}$:

#(2) Stj=cosinePVtjweight→,Phej→.

$S_{tj}$ ranges from −1 to 1, with higher values indicating higher risks of the phenotype.

### Baseline methods and evaluation metrics

We considered 4 baseline methods, (1) a LASSO regression model, (2) a random forest classifier, (3) a gradient boosted tree classifier, and (4) P2V without weights. For LASSO and random forest, we used the Python library “scikit-learn” and set the LASSO regularization strength *C* = 1.0 and the number of trees in random forest *n* = 500 with Gini impurity as the split criterion. For the gradient boosted tree, we used the Python library “xgboost” with the number of rounds *n* = 100. For LASSO, random forest, and gradient boosted tree, we constructed a data matrix with rows representing patients and columns representing counts of medical concepts from past admissions before the latest visit.

We evaluate model performance using area under the receiver operating characteristic curve (AUC-ROC), max F_1_-score, and area under the precision-recall curve (AUC-PR). An ROC curve is created by plotting the true positive rate (also called sensitivity or recall) and false positive rate (1-specificity) at various discrimination thresholds to illustrate the prediction ability of a binary classifier. In general, an AUC-ROC of 0.5 suggests the classifier is uninformative and assigns labels randomly. PR curves are similar to ROC curves, with precision and recall as the axes. A random classifier has an AUC-PR (also called average precision) equal to the percentage of positive samples, that is, the percentage of cases $p_{\mathrm{case}}$ for a phenotype. F_1_-score is the harmonic mean of precision and recall $F_{1}=2\times \frac{\mathrm{precision}\times \mathrm{recall}}{\mathrm{precision}+\mathrm{recall}}$. A dummy classifier that identifies all samples as cases would have a F_1_-score = $\frac{2p_{\mathrm{case}}}{p_{\mathrm{case}}+1}$. A discrimination threshold is needed to calculate the F_1_-score. Since different methods might have different optimal thresholds for different phenotypes, we compute the maximum F_1_-score across all possible discrimination thresholds for each method.

### Phenome-wide predictions using the MIMIC-III database

#### MIMIC-III data preprocessing

We conducted a phenome-wide prediction using the MIMIC-III database and aim to predict incidence risks of phenotypes across the phenome at the latest visit using past medical records. MIMIC-III is a freely accessible critical care database.^23^ We used medical concepts from 3 clinical domains, diagnoses history (ICD-9 codes), prescriptions, and lab tests. There are 46 520 patients in MIMIC-III, among which 39 001 had only 1 admission and 7519 had ≥2 admissions. There are in total 58 951 admissions with 6984 ICD-9 codes, 3267 prescriptions, and 710 lab tests. For prescriptions and lab tests, we used binary information of whether a patient ever had been prescribed a specific drug and whether a patient ever had a specific lab test during an admission. With phecode mapping, the 6984 ICD-9 codes were mapped to 1693 phenotype codes in **Step 1** of PheW^2^P2V. To have good numeric representations of medical concepts using word2vec, we removed rare medical concepts who appeared in fewer than 50 admissions. Similar procedures were taken by other studies with medical concept representations using word2vec.^17^ After these steps, we have 956 phenotype codes, 1348 prescriptions, and 490 lab tests. We used these medical concepts to construct patient sequences, which are medical concepts sequences sorted by admissions. We assume orders of medical concepts from 1 admission do not affect predictions. Therefore, we randomly shuffled medical concepts within 1 admission, and used a window size 500 (a hyperparameter in word2vec to define the maximum distance between the current word and its context word within a sentence) because the maximum number of concepts within 1 admission in MIMIC-III is 497. Table 1 summarizes patients and medical concepts in the MIMIC-III data after processing.

**Table 1.** Summary of the MIMIC-III database after data processing.

		MIMIC-III database
Admissions		58 951
Unique patients		46 520
Patients with only 1 admission		39 001
Patients with at least 2 admissions		7519
Unique medical concepts excluding rare ones	Phenotype codes	956
	Prescriptions	1348
	Lab tests	490
Phenotype codes for predictions (prevalence ≥0.05%)		942

#### Incident cases identification for phenome-wide predictions

Our goal is to predict patients’ incidence risks of phenome-wide phenotypes at the latest visit using patients’ medical history from past visits. We define incident cases at the latest visit as patients who (1) had at least 2 visits, (2) were identified as cases of a phenotype at the latest visit, and (3) had never been identified as the case of the phenotype in past visits. Valid controls are patients who met the conditions (1) and (3) and were identified as controls of the phenotype at the latest visit. For each phenotype, incident cases and valid controls are labeled subjects, while other patients are unlabeled subjects (including patients with one visit, and patients being neither incident cases nor valid controls) which can be used in medical concept embeddings. We calculated phenotype prevalence as the percent of incident cases among all labeled subjects at the latest visit. Among 956 phenotype codes, 14 have prevalence less than 0.05% and were removed from phenome-wide predictions. We predicted the rest 942 phenotypes (Table 1).

**Figure 2.**
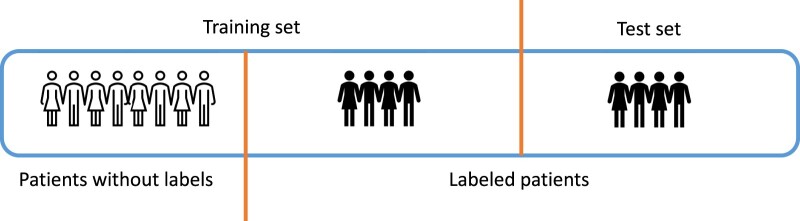
MIMIC-III sample splitting procedures for training and test samples.

For each phenotype, we split labeled subjects with 50% as training data and 50% as test data (Figure 2). Training samples included patients without labels and 50% of labeled patients, while test samples are the other 50% labeled patients (Figure 2). We repeated the random training/test split 10 times to obtain average AUC-ROC, AUC-PR, and max F_1_-score in test sets. Note that for different phenotypes, there are different numbers of labeled subjects.

## Results

### Simulation studies

We conducted simulation studies to evaluate the prediction performance of PheW^2^P2V and baseline methods. We considered binary present/absent medical concepts. Specifically, we simulated a population pool of 20 000 patients each with a binary phenotype concept $C_{0}$ and 150 correlated binary medical concepts, including 10 signal predictor concepts ($C_{1},C_{2},\ldots ,C_{10}$) that predict $C_{0}$ and 140 noise concepts ($C_{11},C_{12},\ldots ,C_{150}$), to mimic correlations between medical concepts. Detailed data generation steps were included in Supplementary Materials A (Figure S1). The outcome phenotype concept $C_{0}$ was generated using a logistic model using the 10 signal concepts, where we set the same β coefficients for them and considered different association strengths ranging β from 0.2 to 0.8 with a grid of 0.1. We set the intercept so that the probability of having outcome $C_{0}$ is around 0.5. Therefore, there will be roughly 10 000 cases and 10 000 controls in the population pool of 20 000 patients. As we do not consider temporal information in a patient sequence, we randomly shuffled medical concepts of each patient. To mimic phenotype prevalence in the MIMIC-III database, we set the case-control ratio as 1:19 in both training and test sets to have a phenotype prevalence 5% and randomly sampled 10 cases and 190 controls from the pool of 20 000 patients. We repeated this procedure 1000 times and obtained prediction results from 1000 test sets. We considered other case-control ratios 1:1, 3:7, and 1:9 (with a prevalence of 50%, 30%, and 10%), and included results in Supplementary Materials A.

**Figure 3.**
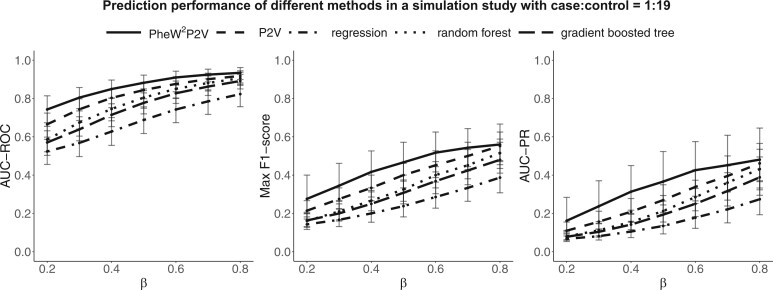
Simulation results of medians, 25th and 75th percentiles of AUC-ROC, max F_1_-score, and AUC-PR of the proposed PheW^2^P2V, the LASSO regression, the random forest classifier, the gradient boosted tree classifier, and the unweighted version P2V with regression coefficient β ranges from 0.2 to 0.8, under the scenario of 1:19 case-control ratio.

We summarized medians, 25th and 75th percentiles of AUC-ROC, max F_1_-score, and AUC-PR for PheW^2^P2V and baseline methods across 1000 test sets in Figure 3. We can see that AUC-ROC, max F_1_-score, and AUC-PR of all 5 methods increase as β increases as expected. PheW^2^P2V outperforms all baseline methods, especially when signals are weak. Results for different case-control ratios were summarized in Supplementary Materials A (Figure S3), where similar patterns were observed. We observed a bigger improvement of PheW^2^P2V over LASSO regression, random forest, and gradient boosted tree for rare phenotypes because the imbalance between cases and controls affects the prediction performance of regression-based and tree-based methods more^25^^,^^26^ than that of P2V methods.

We also conducted simulation studies to demonstrate that medical concept embeddings using word2vec can recover the association strength between a signal medical concept and a phenotype. Results are included in Supplementary Materials A (Figure S2).

### Phenome-wide predictions using the MIMIC-III database

**Figure 4.**
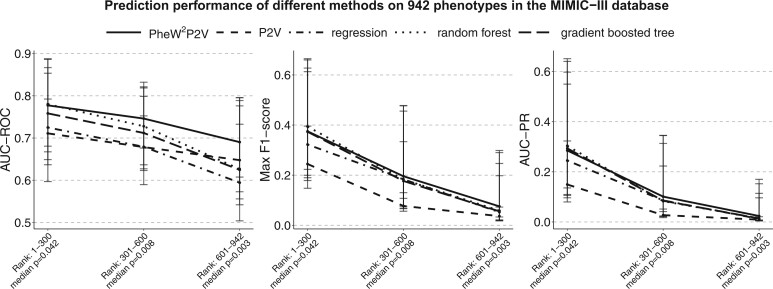
Medians, 25th and 75th percentiles of AUC-ROC, max F_1_-score, and AUC-PR across binned 300 phenotypes with descending prevalence in the MIMIC-III databases for the proposed PheW^2^P2V, the LASSO regression, the random forest classifier, the gradient boosted tree classifier, and the unweighted P2V.

Phenome-wide prediction results across all 942 phenotypes binned with 300 phenotypes ranked by prevalence from the MIMIC-III database was summarized (Table 2) with medians, 25th and 75th percentiles of AUC-ROC, max F_1_-score, and AUC-PR from 10 training/test splits. Across the phenome, PheW^2^P2V has a median AUC-ROC 0.74 (baseline methods have values ≤0.73), a median max F_1_-score 0.20 (baseline methods have values ≤0.19), and a median AUC-PR 0.10 (baseline methods have values ≤0.10). There is a decreasing trend in prediction performance for all methods as phenotypes become rarer as expected. PheW^2^P2V has bigger improvements over baseline methods when phenotypes are rare, which is consistent with simulation results. Results in Table 2 were also plotted in Figure 4 for a better visualization where we can see that the proposed PheW^2^P2V has the highest AUC-ROC, max F_1_-score, and AUC-PR in almost all bins of phenotypes. Table 3 summarizes numbers of phenotypes in the phenome that PheW^2^P2V performs better than baseline methods within each bin of phenotypes. These results clearly demonstrate the advantages of PheW^2^P2V. We also plotted prediction performance for 50 phenotypes randomly selected from each bin in Supplementary Materials A (Figures S6-S8). Full prediction results and descriptions across the phenome of 942 phenotypes are included in Supplementary Materials B.

**Table 2.** Medians and 25th and 75th percentiles of AUC-ROC, max F_1_-score, and AUC-PR of the 942 phenotypes binned by 300 from most to least prevalent phenotypes in the MIMIC-III database.

	Prevalence rank of phenotypes			
	1-300	301-600	601-942	All
**Prevalence median (Q1, Q3)^a^**	0.042 (0.025, 0.075)	0.008 (0.006, 0.011)	0.003 (0.001, 0.003)	0.007 (0.003, 0.024)
**AUC-ROC median (Q1, Q3)^a^**				
PheW^2^P2V	0.78 (0.68, 0.87)	0.75 (0.68, 0.82)	0.69 (0.61, 0.80)	0.74 (0.66, 0.83)
P2V	0.71 (0.65, 0.79)	0.68 (0.63, 0.75)	0.65 (0.57, 0.73)	0.68 (0.62, 0.76)
Regression	0.72 (0.60, 0.85)	0.68 (0.59, 0.80)	0.59 (0.50, 0.78)	0.66 (0.57, 0.81)
Random forest	0.78 (0.67, 0.89)	0.73 (0.64, 0.83)	0.63 (0.56, 0.79)	0.72 (0.61, 0.84)
Gradient boosted tree	0.76 (0.64, 0.89)	0.71 (0.62, 0.82)	0.62 (0.54, 0.80)	0.70 (0.60, 0.84)
**Max F_1_-score median (Q1, Q3)^a^**				
PheW^2^P2V	0.37 (0.22, 0.61)	0.20 (0.11, 0.33)	0.08 (0.03, 0.20)	0.20 (0.09, 0.38)
P2V	0.24 (0.15, 0.40)	0.08 (0.06, 0.13)	0.04 (0.02, 0.07)	0.09 (0.04, 0.20)
Regression	0.32 (0.18, 0.63)	0.18 (0.08, 0.46)	0.05 (0.02, 0.29)	0.19 (0.06, 0.46)
Random forest	0.39 (0.20, 0.66)	0.18 (0.07, 0.48)	0.06 (0.02, 0.25)	0.19 (0.06, 0.50)
Gradient boosted tree	0.37 (0.19, 0.66)	0.18 (0.07, 0.48)	0.06 (0.02, 0.30)	0.19 (0.06, 0.50)
**AUC-PR median (Q1, Q3)^a^**				
PheW^2^P2V	0.28 (0.14, 0.55)	0.10 (0.04, 0.22)	0.02 (0.01, 0.10)	0.10 (0.03, 0.27)
P2V	0.15 (0.08, 0.32)	0.03 (0.02, 0.05)	0.01 (0.00, 0.02)	0.03 (0.01, 0.11)
Regression	0.24 (0.10, 0.60)	0.09 (0.02, 0.31)	0.01 (0.00, 0.15)	0.09 (0.02, 0.35)
Random forest	0.30 (0.11, 0.65)	0.08 (0.02, 0.34)	0.01 (0.00, 0.11)	0.10 (0.02, 0.39)
Gradient boosted tree	0.29 (0.11, 0.64)	0.08 (0.02, 0.35)	0.01 (0.01, 0.17)	0.10 (0.02, 0.39)

a Q1 is the 25th percentile and Q3 is the 75th percentile.

**Table 3.** Numbers of phenotypes that PheW^2^P2V performs better than the corresponding baseline method, across all 942 phenotypes binned by 300 from most to least prevalent in the MIMIC-III database.

	Rank of phenotypes by prevalence			
	1-300	301-600	601-942	All
**Prevalence median (Q1, Q3)^a^**	0.042 (0.025, 0.075)	0.008 (0.006, 0.011)	0.003 (0.001, 0.003)	0.007 (0.003, 0.024)
**AUC-ROC**				
PheW^2^P2V better than P2V	291	289	283	863
PheW^2^P2V better than regression	254	232	249	735
PheW^2^P2V better than random forest	150	183	210	543
PheW^2^P2V better than gradient boosted tree	191	206	215	612
**Max F_1_-score**				
PheW^2^P2V better than P2V	291	282	282	855
PheW^2^P2V better than regression	234	160	186	580
PheW^2^P2V better than random forest	145	162	177	484
PheW^2^P2V better than gradient boosted tree	178	157	172	507
**AUC-PR**				
PheW^2^P2V better than P2V	273	282	279	834
PheW^2^P2V better than regression	217	170	199	586
PheW^2^P2V better than random forest	131	165	181	477
PheW^2^P2V better than gradient boosted tree	148	165	178	491

a Q1 is the 25th percentile and Q3 is the 75th percentile.

### Examples of clinical disease phenotype predictions in the MIMIC-III database

We investigated individual phenotypes to understand the clinical significance of PheW^2^P2V for phenome-wide predictions and highlighted 5 phenotypes from 2 different clinical categories with their prediction performance in Table 4. The first category includes common medical conditions that are potentially preventable or treatable, such as atherosclerosis (phenotype code: 440) and diabetic retinopathy (phenotype code: 250.7). These disorders are frequently under-diagnosed and under-treated despite the fact that effective preventive and therapeutic strategies exist.^27^^,^^28^ PheW^2^P2V has the best prediction performance for these conditions. For example, for diabetic retinopathy, PheW^2^P2V has an AUC-ROC 0.957 (baseline methods have values ≤0.943) and a max F_1_-score 0.524 (baseline methods have values ≤0.515), suggesting that PheW^2^P2V has the potential to identify high risk patients with diabetic retinopathy. The second category includes rare disorders that may be diagnostically challenging, and thus may be missed if not considered in the differential diagnosis. Here, we considered chronic pericarditis (phenotype code: 420.22), meningitis (phenotype code: 320), and aneurysm of iliac artery (phenotype code: 442.2). PheW^2^P2V has superior prediction performance for these conditions. For example, for chronic pericarditis, PheW^2^P2V has an AUC-ROC 0.825 (baseline methods have values ≤0.786) and a max F_1_-score 0.271 (baseline methods have values ≤0.194), suggesting that PheW^2^P2V can help diagnose this rare disorder. These examples demonstrate that PheW^2^P2V is powerful in phenome-wide predictions and is capable of providing clinically-relevant data-driven risk stratification that could be useful as a screening tool to identify and flag patients with high-risk conditions in early stages which may be missed overwise. Note that studies have observed that prediction tools usually have low F_1_-scores to predict rare outcomes,^29–31^ which is also observed in our simulation studies with different case-control ratios summarized in Supplementary Materials A.

**Table 4.** AUC-ROC, max F_1_-score, and AUC-PR of 5 highlighted clinical disease phenotypes in the MIMIC-III database. PheW^2^P2V outperforms all baseline methods across all three metrics.

**Category** ^a^	Disease phenotypes	Prevalence	PheW^2^P2V	P2V	Regression	Random forest	Gradient boosted tree
	**AUC-ROC (std err)**						
I	Atherosclerosis	0.052	**0.862 (0.004)**	0.806 (0.005)	0.780 (0.005)	0.840 (0.003)	0.839 (0.005)
I	Diabetic retinopathy	0.025	**0.957 (0.002)**	0.911 (0.003)	0.864 (0.009)	0.943 (0.002)	0.934 (0.003)
II	Chronic pericarditis	0.007	**0.825 (0.015)**	0.786 (0.018)	0.759 (0.016)	0.734 (0.027)	0.784 (0.014)
II	Meningitis	0.004	**0.833 (0.018)**	0.802 (0.020)	0.612 (0.009)	0.694 (0.016)	0.689 (0.014)
II	Aneurysm of iliac artery	0.002	**0.890 (0.020)**	0.844 (0.019)	0.847 (0.012)	0.833 (0.022)	0.857 (0.010)
	**Max F_1_-score (std err)**						
I	Atherosclerosis	0.052	**0.449 (0.006)**	0.262 (0.004)	0.352 (0.006)	0.447 (0.006)	0.391 (0.007)
I	Diabetic retinopathy	0.025	**0.524 (0.008)**	0.335 (0.007)	0.510 (0.009)	0.515 (0.005)	0.513 (0.006)
II	Chronic pericarditis	0.007	**0.271 (0.017)**	0.130 (0.013)	0.194 (0.021)	0.190 (0.016)	0.188 (0.026)
II	Meningitis	0.004	**0.158 (0.011)**	0.109 (0.005)	0.047 (0.005)	0.048 (0.005)	0.050 (0.010)
II	Aneurysm of iliac artery	0.002	**0.320 (0.067)**	0.087 (0.013)	0.267 (0.036)	0.294 (0.023)	0.269 (0.041)
	**AUC-PR (std err)**						
I	Atherosclerosis	0.052	**0.363 (0.006)**	0.168 (0.004)	0.250 (0.006)	0.337 (0.007)	0.310 (0.005)
I	Diabetic retinopathy	0.025	**0.470 (0.014)**	0.209 (0.004)	0.420 (0.009)	0.420 (0.007)	0.435 (0.014)
II	Chronic pericarditis	0.007	**0.135 (0.017)**	0.048 (0.008)	0.094 (0.015)	0.073 (0.012)	0.083 (0.021)
II	Meningitis	0.004	**0.065 (0.007)**	0.046 (0.008)	0.010 (0.001)	0.012 (0.001)	0.013 (0.002)
II	Aneurysm of iliac artery	0.002	**0.185 (0.065)**	0.023 (0.004)	0.150 (0.031)	0.156 (0.022)	0.106 (0.021)

a Category I includes common conditions amenable to screening and prevention, and Category II includes rare and diagnostically challenging conditions. These categories were selected to illustrate potential clinical utility of PheW^2^P2V predictions.

### Computation time

To show the computational efficiency of PheW^2^P2V on a phenome-wide prediction task, we compared computation time of PheW^2^P2V to that of random forest and gradient boosted tree. PheW^2^P2V is trained once and used for prediction across a phenome with *N* phenotypes with a total computation time: training time + N*prediction time. Random forest and gradient boosted tree are trained individually for each phenotype with a total computation time for the phenome: N*(training time + prediction time). With a machine of Intel(R) Xeon(R) Gold 6226R CPU @ 2.90GHz, a phenome-wide predictions took 91 + 3*N seconds for PheW^2^P2V, 5*N seconds for random forest, and 21*N seconds for gradient boosted tree. PheW^2^P2V will be much faster when predicting a large number of phenotypes *N*.

## Discussion

We developed PheW^2^P2V, a phenome-wide prediction framework that efficiently predicts phenotypes across a phenome by taking a 2-step procedure, that is, medical concept embeddings followed by tailored predictions with a novel weighting scheme. To better define phenome-wide case-control status, PheW^2^P2V maps ICD diagnosis codes to phenotype codes. PheW^2^P2V generates tailored patient vectors for individual phenotypes for tailored predictions. When computing patient vectors, the proposed weighting scheme upweights past medical histories that are most relevant to a phenotype of interest and thus tailors the prediction to the phenotype. The computational efficiency of phenome-wide predictions is achieved by separating embeddings and predictions, making phenome-wide predictions feasible. PheW^2^P2V is fast, flexible, and has better prediction performance than major popular baseline methods consistently across most of the 942 phenotypes in the MIMIC-III database. PheW^2^P2V takes advantages of the word2vec algorithm to numerically represent patients’ medical concepts which avoids imputing missing concepts to convert patients’ medical concepts to a sparse data matrix that is needed by most conventional supervised learning methods. Note that PheW^2^P2V does not use labeled data to directly link predictors and outcomes but uses both labeled and unlabeled data for embeddings. This helps reduce the overfitting problem of traditional supervised learning methods, and overcomes the problem of limited labeled data, especially for rare phenotypes. Thus, PheW^2^P2V performs much better than baseline methods for rare phenotypes. We demonstrated several clinical examples in which PheW^2^P2V can predict less-common conditions that could be diagnostically challenging or missed on a routine clinical work up, such as chronic pericarditis. Therefore, PheW^2^P2V could be useful as a screening tool to prospectively identify and flag patients with high risks conditions in early stages which may be missed otherwise, such as early atherosclerosis, which is preventable with medications and lifestyle changes. We conducted additional experiments using MIMIC-III and investigate the performance of PheW^2^P2V as a screening tool. Results in Supplementary Materials A (Tables S2 and S3, Figures S4 and S5) suggest that the proposed PheW^2^P2V is a better screening tool.

With extensive simulation studies, we demonstrated superior prediction performance of PheW^2^P2V over 4 baseline methods: LASSO regression, random forest, gradient boosted tree, and unweighted P2V. We also demonstrated that numeric vectors of signal medical concepts and outcome concepts can recover association strengths of both directions between them (Supplementary Materials A). We further investigated the prediction performance of PheW^2^P2V using only medical concepts that are positively associated with phenotypes using MIMIC-III and observed a worse prediction performance (Supplementary Materials A—Table S1). This is promising as it suggests that information is preserved through embedding. In contrast to traditional supervised learning methods, patients without labels are informative and can be used to train numeric representations of medical concepts. This advantage enabled us to leverage 39 001 additional patients with only one admission in the MIMIC-III database to conduct the phenome-wide predictions. However, the transferability of medical concept embeddings from 1 EHR database to another need to be studied further.

## Conclusion

In summary, PheW^2^P2V is the first phenome-wide prediction framework. We demonstrated its superior prediction performance and computational efficiency using simulation studies and clinical applications on phenome-wide prediction tasks using the MIMIC-III database. Several showcases of clinical phenotypes suggested great potentials of PheW^2^P2V to serve as a computable predictive tool that can aid in clinical decisions through phenome-wide predictions in real-life clinical settings.

## Supplementary Material

ooae084_Supplementary_Data

## Data Availability

The MIMIC-III database is freely available, upon a credentialing process provided on PhysioNet. Detailed information can be found at https://mimic.mit.edu/docs/gettingstarted/.

## Code availability

All code for the method and visualization presented in this manuscript is available at GitHub https://github.com/jiaguo1000/PheW2P2V.
