# Supplementary material for: PheW2P2V: a phenome-wide prediction framework with weighted patient representations using electronic health records
Source: JAMIA Open. 2024 Sep 14;7(3):ooae084. doi: 10.1093/jamiaopen/ooae084 (PMC11401611; doi:10.1093/jamiaopen/ooae084)
Supplement: ooae084_Supplementary_Data [file ooae084_supplementary_data.zip › 20240730_supplement_A.docx]

**PheW^2^P2V: a Phenome-Wide prediction framework with Weighted Patient representations using electronic health records**

**Supplementary Materials A**

Jia Guo^1^, Krzysztof Kiryluk^2^, Shuang Wang^1*^

1. Department of Biostatistics, Mailman School of Public Health, Columbia University
2. Department of Medicine, Division of Nephrology, Columbia University

* To whom correspondence should be addressed. Tel: +1 212 342 4165; Fax: +1 212 305 9408; Email: [sw2206@columbia.edu](mailto:sw2206@columbia.edu)

**1. SIMULATION STUDIES**

**1.1. Details of simulation settings**

We conducted simulation studies to evaluate the prediction performance of PheW^2^P2V and that of comparison methods. In specific, we simulated a population pool of 20,000 patients each with a binary phenotype concept $C_{0}$ and 150 unique binary medical concepts, including 10 signal predictor concepts (denoted as $C_{1},C_{2},\ldots,C_{10}$) that predict $C_{0}$ and 140 noise concepts (denoted as $C_{11},C_{12},\ldots,C_{150}$). Those 150 binary concepts were generated to be correlated with each other, to mimic potential correlations between medical concepts. To do so, for each patient, we first generated 150 continuous concepts $\tilde{C_{1},}\ldots,\tilde{C_{150}}$ from a multivariate normal distribution with mean 0 and a covariance matrix, where we set the 10 signal concepts to be correlated with $\rho_{ij}=0.6$, the 140 noise concepts to be correlated with $\rho_{ij}=0.05$, and $\rho_{ij}=0.05$ between signal and noise concepts. We then applied the “nearestSPD” algorithm [Higham 1988] to find the nearest positive-definite matrix as the covariance matrix. After obtaining 150 continuous concepts, to mimic real EHR patient medical concept sequences, we median-dichotomized them into binary concepts with 1 indicating a medical concept is recorded. Then, the outcome phenotype concept $C_{0}$ was generated using a logistic model with the 10 signal predictor concepts. Other steps are included in the main text. **Figure S1** displays the steps of simulation studies.

**Figure S1.** Steps of simulation studies.

**1.2. Numeric representations can recover the association strength**

With the same simulation settings, we investigated whether numeric representations of medical concepts using word2vec are able to recover the association strength between a medical concept (i.e., an explanatory predictor) and a phenotype (i.e., an outcome). Given a training set with 100 cases and 100 controls, we performed word2vec to obtain numeric representations for all 151 concepts (10 signal predictor concepts, 140 noise concepts, and 1 outcome concept). We then calculated cosine similarities between the vector of outcome concept $\vec{C_{0}}$ and vectors of signal/noise concepts $\vec{C_{k}}(k=1,\ldots,150)$, and obtained their medians, 25^th^ and 75^th^ percentiles across 1,000 training sets.

**Figure S2** displays the results when association signal strengths range $\beta$ from -1.0 to 1.0. We can see that as the effect size increases, i.e., when absolute values of $\beta$ for signal concepts in logistic regressions increase, absolute values of cosine similarities between numeric vectors of signal concepts and the outcome concept also increase (**Figure S2A**), while cosine similarities between vectors of noise concepts and that of the outcome concept are close to 0 (**Figure S2B**). These results demonstrate that numeric representations of medical concepts preserve the original association signal strength nicely.

It is worth noting that a medical concept that is positively associated with an outcome in a logistic regression model (i.e., with a positive $\beta$ coefficient) is also positively correlated with the outcome when being evaluated using numerically represented vectors (i.e., with a positive cosine similarity). This is expected, because according to word2vec, a positive cosine similarity will be observed between two numeric vectors for two concepts (e.g., $C_{1}$ and $C_{0}$) when they have similar contexts, i.e., when nearby concepts of $C_{1}$ and nearby concepts of $C_{0}$ are similar. When the signal concept $C_{1}$ is positively associated with the outcome concept $C_{0}$, the probability of having outcome $C_{0}$ when concept $C_{1}$ exists is high, i.e., $C_{1}$ and $C_{0}$ usually appear simultaneously in a patient’s medical concept sequence. As a result, $C_{1}$ and $C_{0}$ will have similar contexts which result in a positive correlation between their numeric vectors. Similar explanation goes to scenarios when $C_{1}$ and $C_{0}$ are negatively associated with a negative $\beta$ coefficient, their numeric vectors will be negatively correlated. We thus used positive $\beta$ coefficients in the following simulation studies to evaluate prediction performance of PheW^2^P2V.

**Figure S2.** Simulation results of medians and 25^th^ and 75^th^ percentiles of cosine similarities between vectors of 10 signal concepts (A), vectors of 140 noise concepts (B) and vector of the outcome concept.

**1.3. Simulation studies with imbalanced case/control ratios**

In addition to the 1:19 case-control ratios in the main text, we also considered unbalanced simulation scenarios ranging case/control ratios from 1:1, 3:7, 1:9 to 1:19, with other simulation settings unchanged. We summarized medians, 25^th^ and 75^th^ percentiles of AUC-ROC, max F1-score, and AUC-PR across 1,000 test sets in **Figure S3**. We observed that the improvement of PheW^2^P2V over LASSO regression, random forest, and gradient boosted tree increases as the case-control design becomes more unbalanced. This is because the imbalance affects the prediction performance of regression-based models like LASSO and tree-based models more.

**Figure S3.** Simulation results medians and 25^th^ and 75^th^ percentiles of AUC-ROC, max F1-score, and AUC-PR of the proposed PheW^2^P2V, the LASSO regression, the random forest classifier, the gradient boosted tree classifier, and the unweighted version P2V, with different case-control ratios of 1:1, 3:7, 1:9 and 1:19.

**2. ADDITIONAL STUDIES ON MIMIC-III DATABASE**

**2.1. Negative correlations**

We believed that a negative correlation between a phenotype and a concept is a meaningful association signal for prediction. Hence, we conducted additional experiments to illustrate this using the MIMIC-III database. Specifically, for each phenotype to be predicted, we only kept the codes that are positively correlated with the phenotype and weighted them using the cosine similarity between the embedding of these codes and the embedding of the phenotype, but set 0 weight to codes that are negatively correlated with the phenotype. We can see that the prediction performance is worse than that of the proposed PheW^2^P2V (**Table S1**) that use signals of both directions.

**Table S1.** Prediction performance of a modified version of the proposed PheW^2^P2V, where weights are set to 0 for concepts that are negatively correlated with phenotypes.

|  | **Prevalence rank of phenotypes** | | | |
| --- | --- | --- | --- | --- |
|  | **1-300** | **301-600** | **601-942** | **All** |
| **Prevalence median**  **(Q1, Q3)** | 0.042  (0.025, 0.075) | 0.008  (0.006, 0.011) | 0.003  (0.001, 0.003) | 0.007  (0.003, 0.024) |
| **AUC-ROC median (Q1, Q3)** |  |  |  |  |
| PheW^2^P2V | 0.78 (0.68, 0.87) | 0.75 (0.68, 0.82) | 0.69 (0.61, 0.80) | 0.74 (0.66, 0.83) |
| PheW^2^P2V non-negative | 0.75 (0.68, 0.85) | 0.71 (0.64, 0.78) | 0.64 (0.57, 0.72) | 0.70 (0.62, 0.79) |
| **Max F_1_-score median (Q1, Q3)** |  |  |  |  |
| PheW^2^P2V | 0.37 (0.22, 0.61) | 0.20 (0.11, 0.33) | 0.08 (0.03, 0.20) | 0.20 (0.09, 0.38) |
| PheW^2^P2V non-negative | 0.32 (0.18, 0.55) | 0.11 (0.06, 0.18) | 0.03 (0.02, 0.07) | 0.11 (0.04, 0.28) |
| **AUC-PR median (Q1, Q3)** |  |  |  |  |
| PheW^2^P2V | 0.28 (0.14, 0.55) | 0.10 (0.04, 0.22) | 0.02 (0.01, 0.10) | 0.10 (0.03, 0.27) |
| PheW^2^P2V non-negative | 0.23 (0.10, 0.50) | 0.04 (0.02, 0.09) | 0.01 (0.00, 0.02) | 0.05 (0.01, 0.17) |

**2.2. PheW^2^P2V as a screening tool**

We conducted experiments using the MIMIC-III database to investigate the performance of PheW^2^P2V as a screening tool. Specifically, we calculated sensitivities (**Table S2**) which are plotted in **Figure S4** and specificities (**Table S3**) which are plotted in **Figure S5**, of predicting phenome-wide phenotypes across different thresholds. Note that for PheW^2^P2V and P2V using cosine similarity between a patient vector and the embedding of a phenotype for prediction, thresholds of cosine similarities range from -1 to +1. For Regression and tree-based methods using predicted probability for prediction, thresholds of predicted probabilities range from 0 to 1.

As expected, we observed sensitivities and specificities drop as thresholds increase and it drops faster for phenotypes with lower prevalence. This suggested that different thresholds maybe applied to screen different phenotypes, and this is true for all methods investigated. With increasing thresholds for PheW^2^P2V and P2V, we observed that sensitivity/specificity curves for all three categories of phenotypes with different prevalence drop slower than that of Regression and tree-based methods (**Figures S4** and **S5**). For example, for rare phenotypes ranking 601-942, sensitivities of Regression and tree-based models are close to zero even with a very small threshold of predicted probabilities such as 0.1. While sensitivities of PheW^2^P2V decrease much slower with increased threshold on cosine similarities.

**Table S2**. Medians of sensitivities for different groups of phenotypes, which are ranked and binned by 300 from most to least prevalent in the MIMIC-III database.

|  |  | **Thresholds of cosine similarity** | | | | | | | | | | |
| --- | --- | --- | --- | --- | --- | --- | --- | --- | --- | --- | --- | --- |
| **Method** | **Rank*** | **-1.00** | **-0.80** | **-0.60** | **-0.40** | **-0.20** | **0.00** | **0.20** | **0.40** | **0.60** | **0.80** | **1.00** |
| PheW^2^P2V | 1-300 | 1.00 | 1.00 | 1.00 | 0.97 | 0.92 | 0.86 | 0.79 | 0.70 | 0.55 | 0.27 | 0.00 |
|  | 301-600 | 1.00 | 1.00 | 1.00 | 0.96 | 0.90 | 0.82 | 0.72 | 0.58 | 0.34 | 0.03 | 0.00 |
|  | 601-942 | 1.00 | 1.00 | 1.00 | 0.98 | 0.91 | 0.80 | 0.65 | 0.42 | 0.11 | 0.00 | 0.00 |
| P2V | 1-300 | 1.00 | 1.00 | 1.00 | 1.00 | 0.98 | 0.80 | 0.40 | 0.09 | 0.00 | 0.00 | 0.00 |
|  | 301-600 | 1.00 | 1.00 | 1.00 | 1.00 | 0.95 | 0.69 | 0.29 | 0.04 | 0.00 | 0.00 | 0.00 |
|  | 601-942 | 1.00 | 1.00 | 1.00 | 1.00 | 0.96 | 0.68 | 0.23 | 0.02 | 0.00 | 0.00 | 0.00 |
|  |  | **Thresholds of predicted probabilities** | | | | | | | | | | |
| **Method** | **Rank*** | **0.00** | **0.10** | **0.20** | **0.30** | **0.40** | **0.50** | **0.60** | **0.70** | **0.80** | **0.90** | **1.00** |
| Regression | 1-300 | 1.00 | 0.45 | 0.36 | 0.30 | 0.25 | 0.22 | 0.19 | 0.16 | 0.13 | 0.09 | 0.00 |
|  | 301-600 | 1.00 | 0.15 | 0.11 | 0.09 | 0.07 | 0.06 | 0.05 | 0.04 | 0.03 | 0.02 | 0.00 |
|  | 601-942 | 1.00 | 0.01 | 0.00 | 0.00 | 0.00 | 0.00 | 0.00 | 0.00 | 0.00 | 0.00 | 0.00 |
| Random  forest | 1-300 | 1.00 | 0.60 | 0.36 | 0.16 | 0.05 | 0.01 | 0.00 | 0.00 | 0.00 | 0.00 | 0.00 |
|  | 301-600 | 1.00 | 0.09 | 0.00 | 0.00 | 0.00 | 0.00 | 0.00 | 0.00 | 0.00 | 0.00 | 0.00 |
|  | 601-942 | 1.00 | 0.00 | 0.00 | 0.00 | 0.00 | 0.00 | 0.00 | 0.00 | 0.00 | 0.00 | 0.00 |
| Gradient  boosted tree | 1-300 | 1.00 | 0.39 | 0.30 | 0.24 | 0.19 | 0.16 | 0.13 | 0.09 | 0.06 | 0.03 | 0.00 |
|  | 301-600 | 1.00 | 0.06 | 0.04 | 0.03 | 0.02 | 0.01 | 0.01 | 0.00 | 0.00 | 0.00 | 0.00 |
|  | 601-942 | 1.00 | 0.00 | 0.00 | 0.00 | 0.00 | 0.00 | 0.00 | 0.00 | 0.00 | 0.00 | 0.00 |

* 942 Phenotypes are ranked by their prevalence, from most to least prevalent.

**Table S3**. Medians of specificities for different groups of phenotypes, which are ranked and binned by 300 from most to least prevalent in the MIMIC-III database.

|  |  | **Thresholds of cosine similarity** | | | | | | | | | | |
| --- | --- | --- | --- | --- | --- | --- | --- | --- | --- | --- | --- | --- |
| **Method** | **Rank*** | **-1.00** | **-0.80** | **-0.60** | **-0.40** | **-0.20** | **0.00** | **0.20** | **0.40** | **0.60** | **0.80** | **1.00** |
| PheW^2^P2V | 1-300 | 0.00 | 0.00 | 0.02 | 0.15 | 0.31 | 0.45 | 0.60 | 0.75 | 0.89 | 0.98 | 1.00 |
|  | 301-600 | 0.00 | 0.00 | 0.02 | 0.15 | 0.31 | 0.47 | 0.62 | 0.78 | 0.94 | 1.00 | 1.00 |
|  | 601-942 | 0.00 | 0.00 | 0.00 | 0.10 | 0.26 | 0.44 | 0.62 | 0.82 | 0.98 | 1.00 | 1.00 |
| P2V | 1-300 | 0.00 | 0.00 | 0.00 | 0.00 | 0.09 | 0.49 | 0.88 | 0.99 | 1.00 | 1.00 | 1.00 |
|  | 301-600 | 0.00 | 0.00 | 0.00 | 0.01 | 0.14 | 0.57 | 0.90 | 0.99 | 1.00 | 1.00 | 1.00 |
|  | 601-942 | 0.00 | 0.00 | 0.00 | 0.00 | 0.11 | 0.56 | 0.91 | 0.99 | 1.00 | 1.00 | 1.00 |
|  |  | **Thresholds of predicted probabilities** | | | | | | | | | | |
| **Method** | **Rank*** | **0.00** | **0.10** | **0.20** | **0.30** | **0.40** | **0.50** | **0.60** | **0.70** | **0.80** | **0.90** | **1.00** |
| Regression | 1-300 | 0.00 | 0.94 | 0.97 | 0.98 | 0.98 | 0.99 | 0.99 | 0.99 | 0.99 | 1.00 | 1.00 |
|  | 301-600 | 0.00 | 0.99 | 1.00 | 1.00 | 1.00 | 1.00 | 1.00 | 1.00 | 1.00 | 1.00 | 1.00 |
|  | 601-942 | 0.00 | 1.00 | 1.00 | 1.00 | 1.00 | 1.00 | 1.00 | 1.00 | 1.00 | 1.00 | 1.00 |
| Random  forest | 1-300 | 0.00 | 0.93 | 0.98 | 1.00 | 1.00 | 1.00 | 1.00 | 1.00 | 1.00 | 1.00 | 1.00 |
|  | 301-600 | 0.00 | 1.00 | 1.00 | 1.00 | 1.00 | 1.00 | 1.00 | 1.00 | 1.00 | 1.00 | 1.00 |
|  | 601-942 | 0.00 | 1.00 | 1.00 | 1.00 | 1.00 | 1.00 | 1.00 | 1.00 | 1.00 | 1.00 | 1.00 |
| Gradient  boosted tree | 1-300 | 0.00 | 0.98 | 0.99 | 0.99 | 0.99 | 1.00 | 1.00 | 1.00 | 1.00 | 1.00 | 1.00 |
|  | 301-600 | 0.00 | 1.00 | 1.00 | 1.00 | 1.00 | 1.00 | 1.00 | 1.00 | 1.00 | 1.00 | 1.00 |
|  | 601-942 | 0.00 | 1.00 | 1.00 | 1.00 | 1.00 | 1.00 | 1.00 | 1.00 | 1.00 | 1.00 | 1.00 |

* 942 Phenotypes are ranked by their prevalence, from most to least prevalent.

**Figure S4.** Medians of sensitivities for different groups of phenotypes, which are ranked and binned by 300 from most to least prevalent phenotypes in the MIMIC-III database.

**Figure S5.** Medians of specificities for different groups of phenotypes, which are ranked and binned by 300 from most to least prevalent phenotypes in the MIMIC-III database.

**Figure S6**. AUC-ROC for 50 randomly selected phenotypes in the bin of 300 phenotypes ranked by prevalence in the MIMIC-III database.

**Figure S7**. Max F1 score for 50 randomly selected phenotypes in the bin of 300 phenotypes ranked by prevalence in the MIMIC-III database.

**Figure S8**. AUC-PR for 50 randomly selected phenotypes in the bin of 300 phenotypes ranked by prevalence in the MIMIC-III database.

**REFERENCE**

Higham, N. J. (1988). Computing a nearest symmetric positive semidefinite matrix. Linear algebra and its applications, 103, 103-118.
